# ANXIETY AND DEPRESSION IN OLDER ADULTS POST DEEPWATER HORIZON OIL SPILL

**DOI:** 10.1093/geroni/igad104.3032

**Published:** 2023-12-21

**Authors:** Mickaela Reed, JoNell Strough

**Affiliations:** West Virginia University, Morgantown, West Virginia, United States; West Virginia University, Morgantown, West Virginia, United States

## Abstract

Previous research has investigated challenges such as scarcity in housing, the loss of life, and the onset of mental health disorders such as PTSD that persist once a person has experienced a man-made disaster. This has been analyzed in populations such as older adults and other vulnerable groups of people. However, little to no consideration has been given to how age, in combination with trauma history, relates to indicators of psychological well-being after disasters. Here, using Socioemotional Selectivity Theory (SST) (Carstensen, 2006) and the Strength of Vulnerability Integration Model (SAVI) (Charles, 2013) we investigated age differences in two indicators of psychological well-being—depression and anxiety—among individuals following the 2010 Deepwater Horizon Oil Spill (DHOS). Specifically, trauma history and personal exposure to the DHOS were tested as potential moderators of age differences in psychological well-being. Residents of the US Gulf Coast region (N=2,508, M age= 57.72 yrs; 60.4% Women) were contacted via telephone and asked to complete the Survey of Trauma, Resilience, and Opportunity among Neighborhoods in the Gulf (STRONG). Regression analyses were used to investigate the roles of age, prior trauma, and DHOS exposure on psychological well-being after controlling for income, education, race, and gender. Implications of the findings for disaster preparedness interventions and theories of psychological well-being across the life span are discussed.

